# Photosynthesis in *Ranunculus asiaticus* L.: The Influence of the Hybrid and the Preparation Procedure of Tuberous Roots

**DOI:** 10.3389/fpls.2019.00241

**Published:** 2019-03-12

**Authors:** Petronia Carillo, Carmen Arena, Giuseppe Carlo Modarelli, Stefania De Pascale, Roberta Paradiso

**Affiliations:** ^1^Department of Environmental, Biological and Pharmaceutical Sciences and Technologies, University of Campania Luigi Vanvitelli, Caserta, Italy; ^2^Department of Biology, University of Naples Federico II, Naples, Italy; ^3^Department of Agricultural Sciences, University of Naples Federico II, Naples, Italy

**Keywords:** rehydration, vernalization, photochemistry, photosynthetic pigments, sugars, amino acids

## Abstract

*Ranunculus asiaticus* L. is a quantitative long-day geophyte, grown in a cold greenhouse for cut flowers and potted plants. Flowering in ranunculus is a complex process, strongly steered by temperature and photoperiodism. Vernalization of rehydrated tuberous roots anticipate sprouting and leaf rosette formation and flowering. It is known that the time for flowering and the sensitivity to cold treatment, in terms of flowering anticipation, varies in numerous hybrids, while no information seems to be available on the influence of hybrids and on the vernalization on the photosynthetic process and primary metabolite profiling. We investigated the influence of two ranunculus hybrids, MDR and MBO, and two preparation procedures of tuberous roots, only rehydration (Control, C) and rehydration followed by vernalization (V), on the photosynthesis and photochemistry of plants grown in a climatic chamber, under a controlled environment. In addition, in MBO plants, in which the vernalization showed the main effects, carbohydrate, amino acid and protein levels were also investigated. In control plants, the response of leaf photosynthesis, to increasing white light, revealed higher photosynthetic activity in MDR than in MBO. The quantum yield of PSII (ϕ_PSII_), electron transport rate (ETR) and non-photochemical quenching (NPQ) did not differ between the two hybrids. The maximal photochemical efficiency (Fv/Fm) was higher in MBO than in MDR and showed a decrease in both hybrids after vernalization. The preparation treatment of propagation material affected the light response of photosynthesis in the two hybrids differently, which increased in plants from vernalized tuberous roots, compared to those from only rehydrated in MBO and decreased in MDR, in accordance to the effects of vernalization observed in leaf photosynthetic pigments. In MBO vernalized tuberous roots, starch was rapidly degraded, and the carbon skeletons used to synthesize amino acids. Control plants of MBO, developed more leaves than those of MDR and a consequent larger plant leaf area. Compared to only rehydration, vernalization of rehydrated tuberous roots increased the plant leaf area in both the hybrids. Compared to the control, vernalized tuberous roots of MBO showed higher concentrations of sucrose and free amino acids, which could act as a long-distance signal promoting floral transition in young leaf primordia.

## Introduction

*Ranunculus asiaticus* L. is a perennial geophyte, from the family *Ranunculaceae* native to the Mediterranean basin and Asia Minor, grown as annual crop for cut flowers and potted plants (De Hertogh, 1996). In the wild Mediterranean environment, tuberous roots of *R. asiaticus* sprout in Autumn, when the first rain rehydrates the dried tissue, and develop a rosette of long-petiole leaves (Horovitz, 1985). Floral induction occurs when six to eight leaves are formed, and flowering lasts from February to May. Plants then begin the dormancy process, as the roots dry and the aerial part wilts and disappears during the summer (Meynet, 1993).

For production of propagation material, the harvested tuberous roots are dehydrated to less than 15% moisture (Meynet, 1993).

*Ranunculus asiaticus* exhibits a low temperature requirement (night/day regime 5–10/12–25°C, optimum day 16°C) and has a medium to high light intensity requirement. It is also considered a quantitative long day plant (Horovitz, 1985). The duration of the vegetative period and the number of flowers vary in the genotypes and are affected by the size of the tuberous root: floral induction is earlier, and the flowers are more numerous in plants from greater tuberous roots compared to those from smaller tuberous roots (Meynet, 1993).

Flowering in ranunculus is a complex process, in which both the thermal history of tuberous roots and the photoperiod play a crucial role. Cold treatments of tuberous roots (vernalization) anticipate sprouting, leaf rosette formation, and flowering (Meynet, 1993; Beruto et al., 2009). This has been ascribed to the need for a cold period to break the summer vegetative dormancy in tuberous roots, as in the natural growth conditions (Kamenetsky et al., 2005).

Vernalization and a short day (SD) stimulate meristematic activity in tuberous roots, increasing the number of buds sprouting, as well as the number of leaves and flowers (Meynet, 1993). Tuberization and flowering show a direct antagonism. In fact, a long day (LD) increases the growth of tuberous roots and anticipates flowering compared to SD, however it reduces the number of flowers as it promotes the growth and development of already formed buds (Ohkawa, 1986; Meynet, 1993). Temperature interacts with the photoperiod: a LD promotes flowering at low temperatures, while being ineffective at high temperatures (Farina et al., 1985).

Vernalization is commonly used to break dormancy in many flower geophytes, as it promotes the starch degradation, increasing the sucrose content (Wang et al., 2018). In addition, dormancy in bulbs is regulated by the balance of abscisic acid (ABA) and gibberellic acid (GA) (Liu et al., 2011). Vernalization activates several metabolic pathways involved in the down regulation of ABA and primes the biosynthesis of active GA forms, releasing dormancy (Wang et al., 2018). However, while in other “bulb” species (e.g., Iris, Lilium and Tulipa), application of GA_3_ can partially replace the effect of vernalization (Moe and Berland, 1986). The effects of exogenous GA_3_ in *R. asiaticus* is controversial, as it anticipated flowering and improved the yield of flowers in some experiments (Hassan et al., 1985), while it did not affect flowering time and reduced yield in others (De Pascale and Scognamiglio, 1998; Mayoli et al., 2009).

In the south of Italy, ranunculus is cultivated in a cold greenhouse from rehydrated tuberous roots that are planted from the end of August to the beginning of September and harvested from the end of November until the beginning of April.

Over the past several years, cultivation of this crop has increased all over the world, also by means of breeding and the development of new hybrids (Hamrick, 2003). Contrary to other wild ranunculus species (Madsen, 1993; Madsen and Brik, 1997), little is known in the literature about the plant physiology of *Ranunculus asiaticus*, and no information seems to be available on its photosynthesis and on the influence vernalization has on the photosynthetic process and on the partitioning of sugars and amino acids within the different plant organs.

The aim of this experiment is to investigate the influence of two hybrids, MDR and MBO, and two preparation treatments of tuberous roots, only rehydration (Control, C) and rehydration, followed by vernalization (V), on the photosynthesis, photochemical efficiency and the photosynthetic pigment contents in plants of *R. asiaticus* L., grown in pots, in a controlled environment. In addition, the MBO hybrid, in which vernalization exerted the strongest effects on pigment contents, leaf development and leaf area, profiling of sugars and amino acids in tuberous roots at three plant phenological stages (planting, leaf rosette, and beginning of flowering), in response to the two preparation procedures, was carried out. Our study is the first to complete an analysis of carbohydrates and free amino acids performed on different tissues of *R. asiaticus*.

## Materials and Methods

### Plant Material, Growth Conditions and Experimental Design

The experiment was carried out at the Department of Biology of the University of Naples Federico II (Naples, Italy).

Plants of two hybrids of *Ranunculus asiaticus* L., MDR (medium earliness) and MBO (early flowering) (Biancheri Creations, Italy), were obtained from dry tuberous roots subjected to two preparation procedures, only rehydration (exposure to 11°C for 24 h in a humid chamber, Control, C) and rehydration followed by vernalization (exposure to 3.5 °C for 10 days, V). Tuberous roots of the most common size for each cultivar were used (4–5 cm for MDR and 3–4 cm for MBO). Four treatments derived by the factorial combination of the two hybrids and the two preparation procedures were compared: MBO-C, MDR-C, MBO-V, and MDR-V.

Plants were grown in pot, on a mixture of perlite and peat (70:30 in vol.), in a climatic chamber under a controlled environment (temperature 20/18°C day/night, relative humidity 60–80%, ambient air CO_2_ concentration 370–400 ppm). The mean values of air temperature and relative humidity (day/night) recorded at the end of the experimental period (70 days, from the beginning of September to the middle of November) were 20.1 ± 0.9/17.7 ± 1.1°C and 60.9 ± 9.8/86.7 ± 7.0%, respectively (Mean Value ± Standard Deviation).

Light was provided by white fluorescent tubes (Sylvania luxline plus -T8, F36W/840, Cool white deluxe, Germany) at a Photosynthetic Photon Flux Density (PPFD) of 200 μmol m^-2^ s^-1^ at the canopy level (16 h photoperiod).

Irrigation was alternated with fertigation (three pulses per week in total). In the nutrient solution, pH and electrical conductivity were kept at 6, 0, and 1.5 dS/m, respectively, and the concentration of the nutrient elements was: (in mM) 12.6 N, 2.9 K, 0.9 P, 4.0 Ca, 1.0 Mg, 1.0 S; (in μM) 38.80 Fe, 45.00 B, 0.60 Cu, 3.80 Zn, 9.10 Mn, 1.00 Mo.

### Sampling and Measurements

#### Net Photosynthesis, Chlorophyll *a* Fluorescence Measurements and Leaf Photosynthetic Pigments

Light response curves and instantaneous values of leaf net photosynthesis (NP) were determined using an Infra-Red Gas Analyzer WALZ HCM-1000 (Walz, Effeltrich, Germany) on fully expanded leaves, in plants at the vegetative stage (leaf rosette, 4th week after planting). Measurements of response curves of NP were carried out after a 10 min acclimation to darkness, increasing PPFD by 0, 50, 100, 250, 500, 1000, 1500, and 2000 μmol m^-2^ s^-1^ using a built-in white halogen lamp, under constant conditions in the leaf chamber (20°C, RH 60%, 400 ppm ambient CO_2_ concentration, air flow rate 600 ml min^-1^).

Contextually to the measurements of NP, chlorophyll *a* fluorescence emission measurements were performed on the same leaves using a portable fluorometer FP 100-MAX-LM equipped with a light sensor (Photon System Instruments, Czechia). Maximal PSII photochemical efficiency was calculated as the ratio of variable to maximal fluorescence (Fv/Fm): Fv/Fm = (Fm-Fo)/Fm, where Fv is the difference between maximal and minimal fluorescence (Fm-Fo). The measurements in the light were carried out under climatic chamber conditions at PPFD of 200 μmol photons m^-2^ s^-1^ at canopy level. The quantum yield of PSII electron transport (ϕ_PSII_) was determined according to Genty et al. (1989). The linear electron transport rate (ETR) was derived by the Krall and Edwards (1992). Non-photochemical quenching (NPQ) was calculated as described by Bilger and Björkman (1990). Measurements of photosynthesis and photochemistry were performed on one leaf per plant, in four plants per combination *Hybrid x Preparation treatment*.

Total chlorophyll (a + b) and total carotenoids (x + c, xanthophylls and carotenes) were analyzed in three samples per plant, on frozen leaf disks grounded in 2 mL of ice-cold 100% acetone (three plants per combination *Hybrid x Preparation treatment*). Samples were centrifuged at 3000 rpm for 5 min, and surnatant was read spectrophotometrically (Cary 100 UV-VIS, Agilent Technologies, Santa Clara, CA, United States) at 470, 645, and 662 nm, according to Sorrentino et al. (2018).

Tuberous root tissues of *R. asiaticus* hybrid MBO, subjected to two preparation procedures, only rehydration (Control, C) and rehydration followed by vernalization (V), were sampled at three different plant phenological stages: presprouted tuberous roots, at planting; leaf rosette, plants at vegetative stage with at least five fully developed leaves (3 weeks after planting); beginning of flowering, plants with primordia of the first flower stem visible (10 weeks after planting).

#### Starch, Soluble Sugars and Amino Acids in Tuberous Roots

Starch and soluble sugars were extracted according to Ferchichi et al. (2018) with some modifications. Frozen powdered samples (50 mg) were suspended in 300 μL of ethanol (98%, v/v), incubated for 20 min at 80 °C in a water bath and centrifuged at 14,000 *g* for 10 min at 4°C. The clear supernatants were separated from the pellets and stored in 1 mL tubes at 4°C. The pellets were then submitted to two subsequent extractions with 150 μL of 80% ethanol (v/v) and 150 μL of 50% ethanol (v/v). Each extraction was followed by an incubation for 20 min at 80°C in a water bath and a centrifugation at 14,000 *g*, for 10 min at 4°C. The supernatants of the first and the two following extractions were pooled and stored at -20°C until analysis. For starch determination, the pellets of the ethanol extraction were heated at 90°C for 2 h in 250 μl of 0.1 M KOH. After cooling the samples in ice, they were acidified to pH 4.5 with 70 μl of 1 M acetic acid. Hundred μL of 50 mM sodium acetate pH 4.8 containing 0.2 U α-amylase and 2 U amyloglucosidase were added to an aliquot of 100 μL of acidified samples and the starch was hydrolizedhydrolyzed at 37°C for 18 h. The samples were vortexed and then centrifuged at 13,000 *g* for 10 min at 4°C and the supernatant containing the glucose derived from hydrolyzed starch was used for measurement. Soluble glucose as well as glucose originating from starch hydrolysis were analyzed enzymatically by a FLX-Xenius spectrophotometer (SAFAS, Monaco) according to Geigenberger et al. (1996). Amino acids were extracted according to Carillo et al. (2012) and measured according to Woodrow et al. (2017).

#### Plant Growth

Plant growth was determined as the number of leaves and the leaf area. The number of leaves was monitored weekly. Plant leaf area was estimated by non-destructive analysis of digital images of leaves with ImageJ software 1,50i version (Wayne Rasband National Institute of Health, United States), on five leaves per plant (five plants per combination *Hybrid x Preparation treatment*) and expressed in cm^2^ per plant.

### Statistical Analysis

The experiment was conducted on 10 plants per combination *Hybrid x Preparation treatment*.

All experimental data were analyzed by two-way analysis of variance (ANOVA) using the SPSS 13 software package ^1^. To compare the means of the treatments for each parameter measured, a Tukey *post hoc* test was performed at a significance level of *P* ≤ 0.05 and *P* ≤ 0.01.

For sugars and amino acids, the principal component analysis (PCA) was conducted using Minitab 16.2.1 statistical software, aimed to extract trends when multiple qualitative variables were used, by formulating new variables correlated to the original ones. The PCA outputs included treatment component scores as well as variable loadings to each selected component (Ciarmiello et al., 2015; Carillo et al., 2019).

The heat map was generated using the https://biit.cs.ut.ee/clustvis/ online program package with Euclidean distance as the similarity measure and hierarchical clustering with complete linkage.

## Results

**FIGURE 1:**
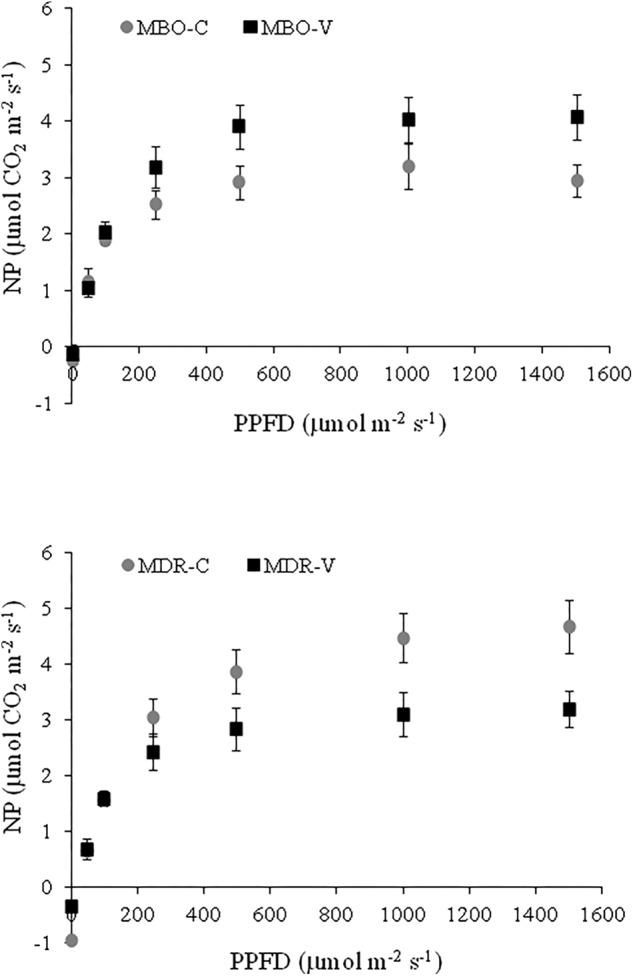
Response curve of leaf net photosynthesis (NP) to increasing white light intensity in plants of *Ranunculus asiaticus* L. hybrids MBO and MDR, obtained by two preparation procedures of tuberous roots, only rehydration (Control, C) and rehydration plus vernalization (V). Plants at vegetative stage (4th week after planting), grown in a climatic chamber under a controlled environment. Measurement conditions in the leaf chamber: 20°C, RH 60%, 400 ppm ambient CO_2_ concentration. Mean values ± standard errors; *n* = 4.

In Control plants of *Ranunculus asiaticus* L., obtained through only rehydrated tuberous roots, the rate of leaf net photosynthesis (NP) increased with the level of white light until a saturating intensity of about 500 μmol m^-2^ s^-1^ in the hybrid MBO and 1000 μmol m^-2^ s^-1^ in MDR was reached (Figure 1). Leaf NP was higher in MDR compared to MBO plants at all the light intensities above 250 μmol m^-2^ s^-1^, with the value of 4.47 μmol CO_2_ m^-2^ s^-1^ at 1000 μmol m^-2^ s^-1^ PPDF vs. 2.92 μmol CO_2_ m^-2^ s^-1^ at 500 μmol m^-2^ s^-1^ PPDF, respectively (Figure 1).

Vernalization of rehydrated tuberous roots affected the response of NP to light intensity differently in the two hybrids of *Ranunculus asiaticus* L. Indeed, this preparation procedure enhanced NP in plants of MBO, while it decreased NP in plants of MDR, at a PPDF above 250 μmol m^-2^ s^-1^, compared to only rehydration (Figure 1).

The PSII maximal photochemical efficiency (Fv/Fm) was higher in MBO than in MDR (0.814 in MBO-C vs. 0.801 in MDR-C) and showed a significant decrease (*P* < 0.05) in both hybrids after vernalization (Table 1). In control plants from only rehydrated tuberous roots, a chlorophyll *a* fluorescence emission analysis evidenced no significant difference in the quantum yield of PSII electron transport (ϕ_PSII_), the linear ETR and the NPQ between the two hybrids, including those with or without vernalization treatment.

**Table 1:** Maximal PSII photochemical efficiency (Fv/Fm), quantum yield of PSII electron transport (ϕ_PSII_), linear electron transport rate (ETR) and non-photochemical quenching (NPQ) in plants of *Ranunculus asiaticus* L. hybrids MBO and MDR, obtained by two preparation procedures of tuberous roots, only rehydration (Control, C) and rehydration plus vernalization (V).

		Fv/Fm	ϕ_PSII_	ETR	NPQ
MDR	C	0.801 ± 0.004	0.756 ± 0.003	38.120 ± 0.171	0.267 ± 0.042
	V	0.789 ± 0.003	0.749 ± 0.008	37.765 ± 0.416	0.165 ± 0.017
*Mean*		*0.796*	*0.753*	*37.943*	*0.217*
MBO	C	0.814 ± 0.003	0.764 ± 0.008	38.545 ± 0.432	0.336 ± 0.048
	V	0.804 ± 0.003	0.767 ± 0.006	38.680 ± 0.338	0.293 ± 0.066
*Mean*		*0.810*	*0.766*	*38.613*	*0.315*
Significance					
Hybrids (H)		^∗^	ns	ns	ns
Preparation treatment (P)		^∗^	ns	ns	ns
H × P		ns	ns	ns	ns

*Plants at vegetative stage (4th week after planting), grown in a climatic chamber under a controlled environment. Mean values ± standard errors; *n* = 4; ns and ^∗^ indicate non-significant or significant at *P* < 0.05, respectively.*

**FIGURE 2:**
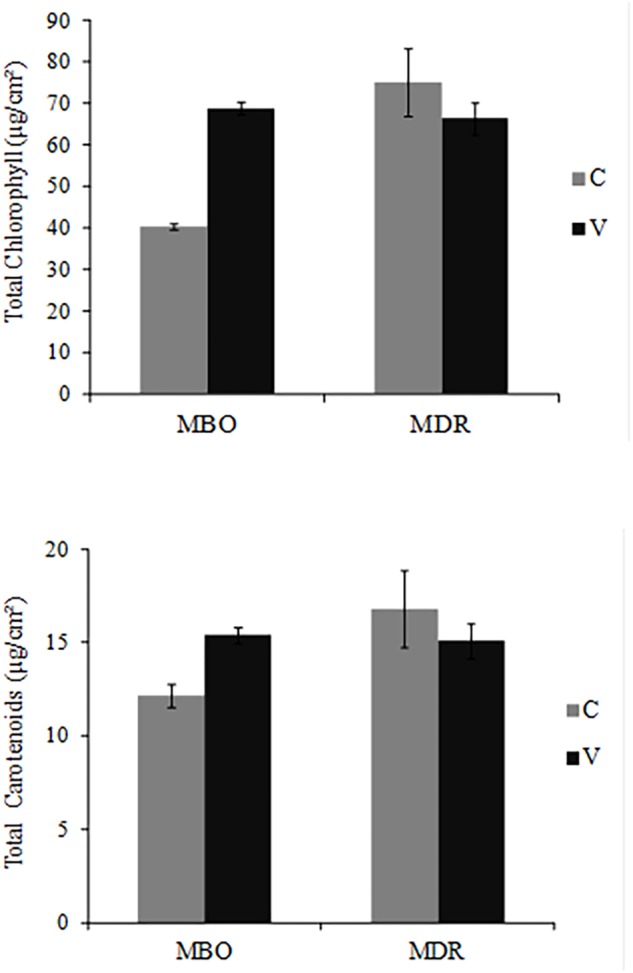
Total chlorophyll (a + b) and total carotenoid (x + c) content in fully expanded leaves of *Ranunculus asiaticus* L. hybrids MBO and MDR, obtained by two preparation procedures of tuberous roots, only rehydration (Control, C) and rehydration followed by vernalization (V). Plants at vegetative stage (4th week after planting), grown in a climatic chamber under a controlled environment. Mean values ± standard errors; *n* = 3.

In the fully developed leaves of control plants, photosynthetic pigment content was higher in MDR than in MBO hybrids, for both total chlorophylls (75.08 μg/cm^2^ vs. 40.42 μg/cm^2^) and total carotenoids (16.80 vs. 8.52 μg/cm^2^) (Figure 2).

**FIGURE 3:**
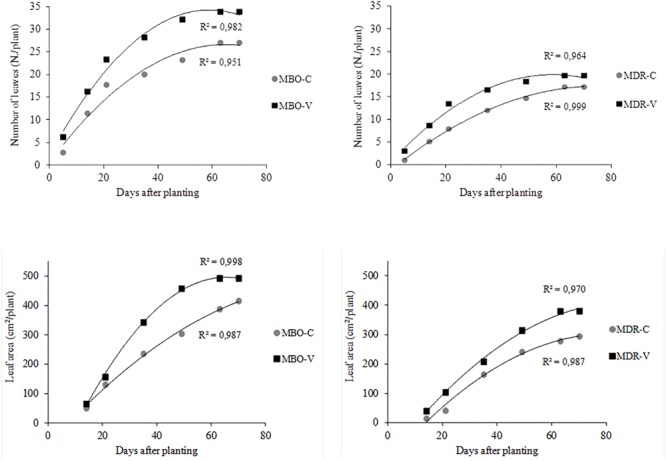
Time evolution of the number of leaves and plant leaf area in plants of *Ranunculus asiaticus* L. hybrids MBO and MDR, obtained by two preparation procedures of tuberous roots, only rehydration (Control, C) and rehydration followed by vernalization (V). Mean values; *n* = 5.

The preparation procedure of tuberous roots affected the synthesis of photosynthetic pigments in the two hybrids differently (Figure 3). More specifically, the pigment amount did not change in the two preparation procedures in MDR, while it was significantly increased by vernalization in MBO (+70% in total chlorophyll and +26% in total carotenoids in MBO-V compared to MBO-C) (Figure 2).

At the complete growth of the aerial part (10th week after planting), in Control plants from only rehydrated tuberous roots, the number of leaves was higher in the MBO compared to the MDR hybrid (27.0 in MBO-C vs. 17.2 leaves per plant in MDR-C). Accordingly, the leaf area was higher in MBO-C than in MDR-C (414.8 vs. 294.0 cm^2^ per plant in MDR-C) (Figure 3).

Plants of the two hybrids showed a different response to the preparation procedure of propagation material in terms of plant growth. In fact, while in MBO vernalization increased both the number of leaves and the leaf area (+7 leaves and +18.5% leaf area per plant, respectively in MBO-V compared to MBO-C) significantly, in MDR it promoted only the leaf expansion (+30% leaf area per plant) (Figure 3).

**FIGURE 4:**
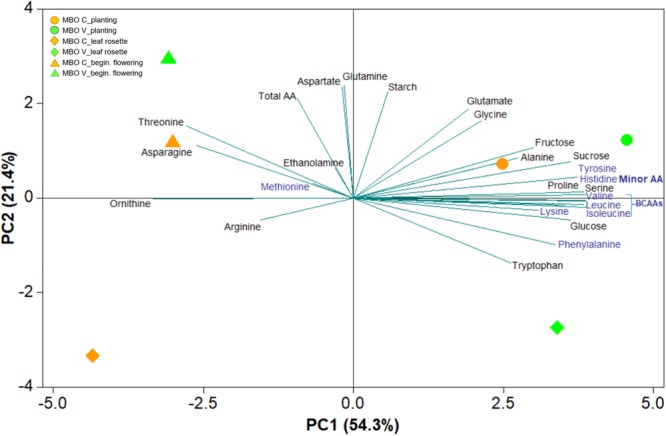
Principal component loading plot and scores of principal component analysis (PCA) of sugars and free amino acids content in tuberous roots at three plant phenological stages (planting, leaf rosette, and beginning of flowering) of *Ranunculus asiaticus* L. hybrid MBO, obtained by two preparation procedures of tuberous roots, only rehydration (Control, C) and rehydration followed by vernalization (V).

In MBO plants, in which vernalization exerted a positive effect on photosynthesis and pigment content, sugars and amino acids were also determined in tuberous roots at three phenological stages (planting, leaf rosette, and beginning of flowering) of *Ranunculus asiaticus* L., in response to the two preparation procedures of tuberous roots, only rehydration (Control, C) and rehydration followed by vernalization (V) (Supplementary Table S1, S2). A comprehensive view of the results of sugars and amino acids in these plant tissues were obtained through the PCA (Figure 4). The first two principal components (PCs) were associated with Eigen values higher than one and explained 75.7% of the cumulative variance, with PC1 and PC2 accounting for 54.3% and 21.4% (Figure 4). PC1 was positively correlated to fructose, glucose, proline, minor amino acids, and was included in branched chain amino acids (BCAAs), serine and alanine. PC1 was negatively correlated to ornithine and threonine, asparagine and arginine. Moreover, PC2 was positively correlated to glutamate, total amino acids, starch, glutamine and aspartate. In the current experiment, the score plot of the PCA superimposed on the above matrix of variables in both tissue x treatments revealed a strong clustering of the tuberous root tissues at planting along the PC1 in the first quadrant, concentrating the sucrose, fructose, part of the minor amino acids, alanine and proline, and the tissues of tuberous roots at initial flowering, in the second quadrant, concentrating the threonine asparagine and total amino acids. The PCA also revealed a strong clustering of MBO-C and MBO-V tuberous roots at the rosette stage, along the negative side of PC2 in the third and fourth quadrants, respectively (Figure 4).

**FIGURE 5:**
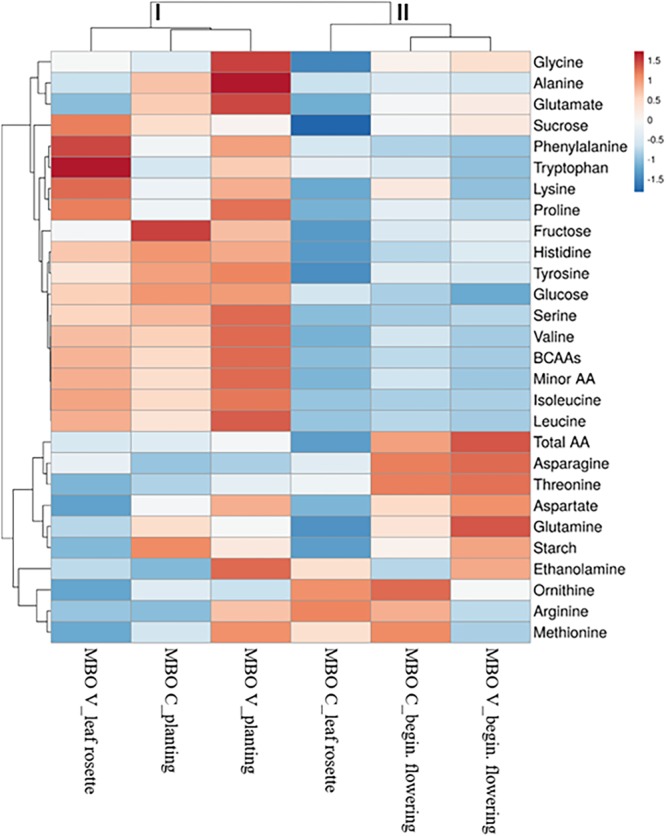
Cluster heat map analysis summarizing sugars and free amino acid changes in tuberous roots at three plant phenological stages (planting, leaf rosette and beginning of flowering) of *Ranunculus asiaticus* L. hybrid MBO, in response to two preparation procedures of tuberous roots, only rehydration (Control, C) and rehydration followed by vernalization (V).

The heat map in Figure 5 provides an integrated view of the responses of sugars and amino acids in MBO tuberous roots, at the three plant phenological stages, to control or vernalization treatment. The heat-map identified two main clusters: I and II (Figure 4). Tuberous roots of MBO-C and MBO-V at planting, and roots of MBO-V at rosette stage, were clustered together in the cluster I and clearly separated from the other three samples present in cluster II. The main clustering factor was the concentration of hexoses (glucose plus fructose), minor amino acids and BCAAs, which were, on average, 3.4, 2.3, and 3.8-fold higher in the samples of cluster I than in those of cluster II, respectively. Glutamate highly contributed to the determination of a sub-cluster in cluster I, since the concentration of glutamate in the two tuberous roots was on average, 2.7-fold higher than in all other samples of tuberous roots. In cluster II, grouping MBO-C at rosette stage and MBO-C and MBO-V at beginning of flowering, minor amino acids included BCAAs, and sucrose drastically decreased, while total amino acids, and in particular asparagine, aspartate and glutamine in tissues at beginning of flowering, increased. In MBO-V at beginning of flowering, there was a 50% and 69% increase in starch and ethanolamine concentration, respectively, compared to MBO-C.

## Discussion

The response curves of leaf net photosynthesis to increasing white light, obtained in plants of *Ranunculus asiaticus* L. at vegetative stage, showed some intrinsic differences between the hybrids, indicating a higher photosynthetic capacity in MDR compared to MBO. In fact, in control plants obtained by only rehydrated tuberous roots, net photosynthesis was higher in MDR compared to MBO plants, at all the light intensities above 250 μmol m^-2^ s^-1^. No data seems to be available in the literature for photosynthesis of *Ranunculus asiaticus* L. In general, the rate of photosynthesis measured in our experiment seems to be lower than that reported for other geophytes (e.g., Lilium), grown in a greenhouse for flower production (Barrera-Aguilar et al., 2013). This result could depend on genetic reasons, but it could also be due to the adaptation of ranunculus plants to the relatively low PPFD applied in the experiment, as well as to the lower photosynthetic capacity known for several species in the young developmental stage (i.e., 4th week after planting) (Kitajima et al., 2002; Paradiso et al., 2018). However, the values recorded for both the hybrids of ranunculus, grown under artificial light in a controlled environment, are in the order of magnitude of those measured in wild plants of the spring tuberous roots species *Anemone raddeana* Regel (Family *Ranunculaceae*; Yoshie and Yoshida, 1987).

The different photosynthesis of the two hybrids seems to be due to a diverse amount of photosynthetic pigments rather than differences in photochemistry. In fact, the fluorescence emission analysis indicates a good performance of the photosynthetic apparatus in a light reaction of photosynthesis, in both hybrids. In our opinion, the intrinsic difference in the photosynthetic pigment content may in part explain the differences recorded in photosynthesis, and in particular, the opposite response to vernalization in the two hybrids for both pigment content and net photosynthesis.

The lower content of photosynthetic pigments in MDR-V compared to MDR-C could also explain the occurrence of differences in maximal PSII photochemical efficiency (Fv/Fm) and net photosynthesis found between the hybrids. In fact, no difference was found between the hybrids in the quantum yield of PSII electron transport (ϕ_PSII_) and in the linear ETR. Conversely, Fv/Fm was lower in MDR-C than in MBO-C, indicating a better condition for the photosystem II and a higher potential capability to convert light energy to reaction centers in the MBO-C hybrid. It is noteworthy that Fv/Fm can be considered as a good indicator of the plant health status; in absence of physiological stress the threshold value is around 0.83 (Björkman and Demmig, 1987). Lower values can provide an indication of unfavorable conditions for photosynthetic apparatus, highlighting a possible photoinhibition under stressful events (Maxwell and Johnson, 2000; Baker and Rosenqvist, 2004). In our case, even if the Fv/Fm was lower in MDR-C compared to MBO-C we can exclude phenomena linked to photosystem photoinhibition, since the Fv/Fm values for both hybrids were close to the optimal value. In both the hybrids, vernalization seems to determine an apparent reduction of NPQ, even if the differences are not statistically significant. It may be hypothesized that this treatment reduces the need for the photosynthetic apparatus to dissipate as heat the light energy was not utilized in photochemistry (Johnson et al., 1993; Horton et al., 1996; Maxwell and Johnson, 2000). It can be supposed that vernalization may likely help photosynthetic apparatus of MBO to withstand the low temperatures. However, further studies on *R. asiaticus* physiology may clarify these aspects.

In our experiment, the increase of pigment content and thus of photosynthesis, observed in MBO-V compared to MBO-C, agrees with studies on herbaceous crops chilled at the pre-sowing seed stage, such as *Foeniculum vulgare* Mill (Rashad and El-Soud, 2009), *Beta vulgaris* L. (Sakr et al., 2013) and *Solanum lycopersicum* L. (Haroun et al., 2011). Our results are also consistent with studies on *Cicer arietinum* L. and *Luffa aegyptiaca* Mill (Singh, 1984) and on *Lolium perenne* L. (Stapleton and Jones, 1987) plants subjected to cold treatment during vegetative development. Conversely, the decrease observed for MDR-V compared to MDR-C is in line with a study on *Glicine max* (L.) Merr seedlings (Yadegari et al., 2008), *Ocimum basilicum* L. plants (Borowski and Blamowski, 2009) and on *Coffea arabica* L. plants (Partelli et al., 2009) exposed to cold treatment. The effect of different *hybrid x tuberous roots preparation procedure* interaction on pigment content and photosynthesis carried out in our study is consistent with some findings in which it has been hypothesized the existence of a different *genotype x abiotic stress* response in various species (Bączek-Kwinta et al., 2007; Bekhradi et al., 2015; Kalisz et al., 2016; Tuteja and Gill, 2016). On the other hand, the different behavior observed in *R. asiaticus* hybrids may also be due to a less pronounced effect of vernalization in MDR, likely suggesting the occurrence of a diverse cold requirement in these hybrids. In addition, the sensitivity to the cold treatment may have been influenced by the different sizes of tuberous roots in the two hybrids: bigger tuberous sizes may likely require a longer vernalization period to obtain the same effect. However, these aspects in *R. asiaticus* remain in part unsolved and further studies are required to confirm these hypotheses.

In plants from rehydrated tuberous roots, the growth in terms of number of leaves and total leaf area per plant differed between the hybrids. Specifically, in the control preparation procedure, MBO plants produced more leaves and reached a greater leaf area compared to MDR, despite the smaller size of the tuberous roots. This result confirms the existence of different vegetative types in *R. asiaticus* and suggests that larger tuberous root sizes are advised for less vigorous genotypes (Meynet, 1974; Beruto et al., 2018).

Compared to the control treatment (only rehydration), vernalization of rehydrated tuberous roots significantly increased the plant leaf area in both the hybrids, in accordance with the promoting effect of low temperature on the meristematic activity known in *R. asiaticus* (Meynet, 1993) and in other bulbous species (Wang et al., 2018).

Sugars and amino acids were analyzed in the MBO hybrid, in which vernalization exerted the strongest effects on photosynthesis and pigment content. These metabolites provide energy and precursors for plant growth and sustain the development of flowers, serving as chemical precursors and energy for floral primary and secondary metabolism (Muhlemann et al., 2014; Borghi and Fernie, 2017).

Vernalization-related cold stress can compromise membrane fluidity and increase oxidation, due to the uncoupling of the electron transport chain (Taïbi et al., 2018). As a consequence, high levels of reactive oxygen species (ROS) are formed, which, in turn, further affect the integrity and functionality of the same membranes and subcellular structures (Thakur and Nayyar, 2013).

In MBO-V tuberous roots, a faster hydrolysis of starch compared to MBO-C, and a strong accumulation of glutamate, alanine, glycine, proline and minor amino acids in addition to glucose and fructose (hexoses), but not sucrose, occurred.

The hexoses, that accumulate in MBO-V tuberous roots after vernalization, can function as osmoprotectants and ROS (hydroxyl radicals) scavengers, maintaining the integrity and stability of membranes and macromolecules (Taïbi et al., 2018). Moreover, the accumulation of minor amino acids can be closely related to the high levels of hexoses, which particularly increase under stress (Fritz et al., 2006). Minor amino acids included BCAAs, which can serve both as compatible compounds, antioxidants and as alternative electron donors for the mitochondrial electron transport chain (Woodrow et al., 2017 and references therein).

It is also likely that the increase of the other amino acids in MBO-V is due to the stressing effects of vernalization. Glutamate, in fact, can serve as precursor of proline, that over and above its supposed role as an osmolyte, can scavenge ROS and buffer cellular redox potential, stabilizing membranes and proteins, and lead to the expression of stress responsive genes containing proline response elements (Carillo, 2018). In addition, glutamate can be converted to GABA, through a decarboxylation catalyzed by glutamate decarboxylase which consumes protons, buffering stress-induced cytosolic acidosis (Carillo, 2018). Subsequently, GABA undergoes a series of reactions, known as GABA shunt, which use alanine as a precursor and supply NADH and/or succinate to the mitochondrial electron-transport chain, under conditions in which respiration and the TCA cycle are impaired and ROS is increased (Bouché et al., 2003). Additionally, glycine, that significantly increases in cold-sensitive seedlings, can be related to the synthesis of glutathione, of which it is a constituent (Taïbi et al., 2018). Glutathione functions not only as a ROS scavenger but also as a cofactor for several enzymes involved in the oxidative stress response (Woodrow et al., 2017).

Furthermore, the increase in ethanolamine in MBO-V tuberous roots, an amino acid derivative obtained by decarboxylation of serine, is useful for the synthesis and/or regeneration of phospholipids, and for changes in the membrane phospholipid profile under cold stress (Kogan et al., 2000).

Glutamate concentration in tuberous roots strongly decreased at the stages of leaf rosette and at the beginning of flowering, in contrast with previous reports (Lea and Forde, 2007; Ishizaki et al., 2010). The decrease of glutamate could depend on its use as a nitrogen donor in biosynthetic transamination in the production of other amino acids. In fact, in MBO-V leaf rosettes, phenylalanine and tryptophan increased while proline was maintained at the same level as in the MBO-V tuberous roots. Phenylalanine and tryptophan are not only essential for protein synthesis in plants, but they are used as precursors for a variety of plant hormones, such as auxin and salicylate, and aromatic secondary metabolites with various biological functions, such as volatile organic compounds (Tzin and Galili, 2010; Muhlemann et al., 2012). In particular, phenylalanine synthesis and accumulation are essential to provide the building blocks for floral enzymes and structural proteins, even in plants containing low amounts of volatile organic compounds (Borghi and Fernie, 2017).

Furthermore, in MBO-C there was a strong increase of two amino acids, arginine and ornithine. The arginine biosynthetic pathway in plants consists of nine enzymatic steps from glutamate, also involving the production of ornithine as an intermediate. These two amino acids can function as precursors of polyamines, which are involved in many pivotal biological processes such as flowering, fruit ripening and osmotic stress protection (Kalamaki et al., 2009). The active role of single types of polyamines in floral development has been reported in several crops, and in particular in long-day plants. The inhibition of the polyamine biosynthetic pathway, preventing flowering in *Spirodela punctata* (G.F.W. Mey.) Thompson and *Polianthes tuberosa*, can be resumed when spermidine, one of the most abundant polyamines, is applied exogenously (Liu et al., 2006 and references therein). However, spermidine has been proven as necessary but not sufficient for promoting flower induction in *Spirodela punctata* (Bendeck de Cantú and Kandeler, 1989). Therefore, the accumulation of arginine and ornithine, precursors of polyamines, can represent a plant strategy to be ready to produce polyamines, which is one of the signals that play a direct role in the promotion of flowering (Kalamaki et al., 2009). The presence of high levels of these two amino acids in tuberous roots of MBO-C, but not in MBO-V, at leaf rosette and beginning of flowering may indicate that in the absence of vernalization the synthesis of metabolites involved in the induction of flowering is delayed.

The signal responsible in MBO-V rosettes, for the use of arginine and ornithine to synthetize polyamines and promote flowering, could be the accumulation of sucrose. It is well known that exogenous sucrose can promote flowering in several species (Bernier et al., 1993). In the juvenile phase, the expression of the florigen gene (FT) is inhibited by miR172-targeted AP2-like transcription factors. miR172 is a conserved miRNA, that is a short, single-stranded non-coding RNA regulating the FT gene expression at a post-transcriptional level. Sucrose is able to inactivate the inhibition of miR172 and promote flowering. In addition, high levels of sucrose induce an increase of trehalose 6 phosphate T6P, which contributes to the production of FT in leaves through a distinct genetic pathway, allowing plant flowering at the correct time when sucrose levels are suitable (Wahl et al., 2013; Yadav et al., 2014).

Finally, at the stage of beginning of flowering, tuberous roots of MBO-V show a strong accumulation of primary amino acids, in particular amides and aspartate, starch and again ethanolamine. The amides asparagine and glutamine, with a high N:C ratio (2:4), can efficiently serve as a carrier and storage metabolite for nitrogen. Increasing the concentration of these two amino acids and aspartate, in addition to starch and ethanolamine can be an effective strategy to store reserves or precursors that can be used during ovule maturation and embryo growth (Borghi and Fernie, 2017).

## Conclusion

In control plants obtained by only rehydrated tuberous roots, the hybrid MDR showed a lower plant leaf area but better efficiency in light interception, and higher net photosynthesis and photosynthetic pigment content compared to MBO. However, the lower photosynthesis in MBO plants was not due to the impairment of photosystems as the Fv/Fm values were comparable in the two hybrids.

Compared to only rehydration, rehydration followed by vernalization had a positive effect in the hybrid MBO, increasing the photosynthetic pigments and the net photosynthesis as well as the plant leaf area. The reasons why vernalization had different effects in the hybrids may be linked to a genotype specific response as well as the different sizes of the tuberous roots, confirming that the size of propagation material should be considered in the selection of both the hybrid and the preparation procedure. Our findings also suggest to perform physiological screenings, in order to define the optimal combination hybrid/vernalization treatment. Moreover, the MBO hybrid, in response to vernalization showed a fine tuning of selected sugars and amino acids in tuberous roots, which prepared plants for flowering. In particular, the concerted action of protectant metabolites in tuberous roots helped the plant to cope with the vernalization related-cold stress, and subsequently in rosette tissues to divert metabolism toward the utilization or accumulation of particular amino acids and sucrose functions to promote flowering.

## Author Contributions

GCM performed plant cultivation and contributed to measurements. RP, CA, PC performed measurements and statistical analysis, and wrote the manuscript. SDP provided scientific oversight in experimental design.

## Conflict of Interest Statement

The authors declare that the research was conducted in the absence of any commercial or financial relationships that could be construed as a potential conflict of interest.

## Footnotes

1 www.ibm.com/software/analytics/spss
