# Supplementary material for: Photosynthesis in Ranunculus asiaticus L.: The Influence of the Hybrid and the Preparation Procedure of Tuberous Roots
Source: Front Plant Sci. 2019 Mar 12;10:241. doi: 10.3389/fpls.2019.00241 (PMC6423076; doi:10.3389/fpls.2019.00241)
Supplement: Supplementary file 1 [file Table_1.docx]

Table S1. Free amino acid content in tuberous roots of *Ranunculus asiaticus* L. hybrids MBO, obtained by two preparation procedures, only rehydration (Control, C) and rehydration plus vernalization (V), at three plant phenological stages: planting, leaf rosette, beginning of flowering. Plants grown in climatic chamber under controlled environment. Mean values ± standard errors; n=3.

|  | Planting | | | Leaf rosette | | | Beginning of flowering | | |
| --- | --- | --- | --- | --- | --- | --- | --- | --- | --- |
|  | MBO-C | MBO-V | *mean* | MBO-C | MBO-V | *mean* | MBO-C | MBO-V | *mean* |
| Alanine | 3.87±0.26 | 5.64±0.37 | *4.76* | 1.52±0.17 | 1.52±0.31 | *1.52* | 1.79±0.41 | 1.60±0.39 | *1.70* |
| Arginine | 0.36±0.05 | 0.91±0.06 | *0.64* | 1.06±0.25 | 0.38±0.11 | *0.72* | 0.97±0.27 | 0.44±0.06 | *0.70* |
| Asparagine | 17.68±0.51 | 21.37±2.28 | *19.53* | 35.35±7.04 | 39.01±0.68 | *37.18* | 85.46±13.97 | 88.53±16.71 | *86.99* |
| Aspartate | 0.69±0.08 | 1.03±0.07 | *0.86* | 0.35±0.04 | 0.29±0.05 | *0.32* | 0.91±0.21 | 1.10±0.18 | *1.01* |
| Ethanolamine | 0.32±0.07 | 0.68±0.03 | *0.50* | 0.55±0.09 | 0.37±0.06 | *0.46* | 0.37±0.08 | 0.62±0.03 | *0.49* |
| Glycine | 0.69±0.03 | 1.11±0.01 | *0.90* | 0.46±0.09 | 0.78±0.18 | *0.62* | 0.80±0.17 | 0.89±0.14 | *0.84* |
| Glutamate | 10.95±0.59 | 15.30±1.02 | *13.13* | 1.42±0.28 | 2.24±0.22 | *1.83* | 7.10±1.02 | 8.62±0.90 | *7.86* |
| Glutamine | 34.49±1.99 | 26.90±2.20 | *30.70* | 7.24±1.71 | 16.67±1.36 | *11.95* | 33.06±8.57 | 47.80±3.17 | *40.43* |
| Isoleucine | 2.74±0.22 | 3.81±0.09 | *3.28* | 0.56±0.13 | 3.39±0.32 | *1.97* | 0.76±0.18 | 0.74±0.15 | *0.75* |
| Histidine | 3.83±0.22 | 3.68±0.18 | *3.76* | 0.99±0.12 | 3.40±0.30 | *2.19* | 1.66±0.22 | 2.03±0.38 | *1.85* |
| Leucine | 2.34±0.12 | 3.64±0.02 | *2.99* | 0.61±0.12 | 2.98±0.34 | *1.80* | 0.84±0.12 | 0.72±0.13 | *0.78* |
| Lysine | 2.28±0.17 | 2.92±0.30 | *2.60* | 1.68±0.25 | 3.18±0.52 | *2.43* | 2.56±0.03 | 1.80±0.34 | *2.18* |
| Methionine | 0.15±0.02 | 0.36±0.03 | *0.25* | 0.28±0.05 | 0.07±0.01 | *0.17* | 0.36±0.04 | 0.12±0.03 | *0.24* |
| Ornithine | 2.14±0.19 | 1.96±0.05 | *2.05* | 3.19±0.82 | 1.57±0.23 | *2.38* | 3.33±0.44 | 2.38±0.35 | *2.85* |
| Phenylalanine | 1.45±0.20 | 2.22±0.05 | *1.83* | 1.16±0.18 | 2.58±0.62 | *1.87* | 0.97±0.18 | 0.87±0.19 | *0.92* |
| Proline | 0.57±0.12 | 1.07±0.08 | *0.82* | 0.24±0.05 | 1.05±0.06 | *0.64* | 0.51±0.00 | 0.36±0.01 | *0.44* |
| Serine | 4.35±0.30 | 5.22±0.27 | *4.79* | 1.26±0.21 | 3.99±0.49 | *2.63* | 1.48±0.20 | 1.69±0.31 | *1.59* |
| Tyrosine | 2.10±0.14 | 2.20±0.31 | *2.15* | 0.81±0.14 | 1.75±0.36 | *1.28* | 1.38±0.10 | 1.26±0.31 | *1.32* |
| Threonine | 1.44±0.07 | 1.98±0.36 | *1.71* | 2.15±0.20 | 1.12±0.40 | *1.64* | 3.62±0.29 | 3.69±0.40 | *3.66* |
| Tryptophan | 1.03±0.09 | 1.93±0.05 | *1.48* | 1.24±0.21 | 2.75±0.58 | *2.00* | 1.09±0.14 | 0.73±0.11 | *0.91* |
| Valine | 4.59±0.47 | 5.98±0.18 | *5.29* | 1.06±0.23 | 4.89±1.19 | *2.97* | 2.12±0.17 | 1.52±0.36 | *1.82* |
| BCAAs | 9.68±0.78 | 13.44±0.26 | *11.56* | 2.23±0.46 | 11.26±1.77 | *6.75* | 3.73±0.47 | 2.98±0.61 | *3.36* |
| Total AA | 98.07±3.24 | 109.95±5.88 | *104.01* | 63.15±11.69 | 93.99±3.84 | *78.57* | 151.15±9.58 | 167.52±17.19 | *159.34* |
| Minor AA | 19.84±1.03 | 25.72±0.52 | *22.78* | 8.20±1.42 | 22.62±3.34 | *15.41* | 11.63±1.08 | 9.51±1.75 | *10.57* |
