# Supplementary material for: Photosynthesis in Ranunculus asiaticus L.: The Influence of the Hybrid and the Preparation Procedure of Tuberous Roots
Source: Front Plant Sci. 2019 Mar 12;10:241. doi: 10.3389/fpls.2019.00241 (PMC6423076; doi:10.3389/fpls.2019.00241)
Supplement: Supplementary file 2 [file Table_2.docx]

Table S2. Starch, glucose, fructose and sucrose content in tuberous roots of *Ranunculus asiaticus* L. hybrids MBO, obtained by two preparation procedures, only rehydration (Control, C) and rehydration plus vernalization (V), at three plant phenological stages: planting, leaf rosette and beginning of flowering. Plants grown in climatic chamber under controlled environment. Mean values ± standard errors; n=3.

|  | Planting | | | Leaf rosette | | | Beginning of flowering | | |
| --- | --- | --- | --- | --- | --- | --- | --- | --- | --- |
|  | MBO-C | MBOV | *mean* | MBO-C | MBOV | *mean* | MBO-C | MBOV | *mean* |
| Starch | 5.91±0.44 | 4.05±0.47 | *4.98* | 0.61±0.08 | 1.16±0.11 | *0.89* | 3.69±1.26 | 5.53±0.23 | *4.61* |
| Glucose | 5.21±0.43 | 5.10±0.39 | *5.15* | 1.64±0.16 | 4.27±0.86 | *2.96* | 1.05±0.55 | 0.25±0.07 | *0.65* |
| Fructose | 5.74±0.23 | 4.33±0.42 | *5.04* | 0.59±0.02 | 2.80±0.36 | *1.70* | 2.10±0.62 | 2.46±0.31 | *2.28* |
| Sucrose | 2.49±0.28 | 2.19±0.29 | *2.34* | 0.87±0.11 | 3.00±0.21 | *1.93* | 2.09±0.34 | 2.36±0.24 | *2.23* |
